# Effect of the COVID-19 Pandemic on Medical Students at the University of Jeddah, Saudi Arabia

**DOI:** 10.7759/cureus.38968

**Published:** 2023-05-13

**Authors:** Abdulrahman Alesawi, Hisham Rizk, Ali Aledrisi, Abdulaziz Alramadhani, Saleh Saber, Mohammed Namenkani

**Affiliations:** 1 Faculty of Medicine, University of Jeddah, Jeddah, SAU; 2 Surgery Department, Faculty of Medicine, University of Jeddah, Jeddah, SAU

**Keywords:** online medical education, medical students, jeddah, pandemic, covid-19

## Abstract

Background

This study aims to identify the coronavirus disease 2019 (COVID-19) pandemic impact on the academic, financial, psychological, and hygienic aspects of medical students at the University of Jeddah.

Methodology

Three hundred fifty medical students from the University of Jeddah were sent an online questionnaire using a simple consecutive type of sampling for this cross-sectional study. Students from the preclinical and clinical years were included. The survey consisted of 39 items: four questions were for the demographic domain, 14 items were for the academic domain, another 14 were for hygienic, psychological, and financial aspects, and seven items assessed the effect on elective. A P-value of less than 0.05 was regarded as significant during the statistical analysis, which was conducted using Statistical Package for Social Sciences (SPSS) version 25 (IBM Corp., Armonk, NY, USA).

Results

There were 333 responses, 174 (52.3%) of them were males. The commonest age group was 21-23 years (n=237, 71.2%). Most of the participants were living in Jeddah (n=307, 92.2%). The majority (54%, n=180) agreed or strongly agreed that “Fluctuations in lecture timing is one of the downsides of online teaching” and “The hands-on experience has suffered greatly” and 42% (n=140) strongly agreed on "Technical issues like poor WiFi connection and lack of computer or mobile devices make online teaching difficult”. One hundred five (31.5%) of the participants had an elective during the pandemic, of which 41 (39%) did not have it in training centers. In terms of the mental aspect, 154 (46.2%) students were impacted by the COVID-19 pandemic, and 111 (72.1% of them) developed anxiety or depression. Social media (n=150, 45%) was the most preferable source of information during the pandemic.”.

Conclusion

The COVID-19 pandemic affected the academic advancement of medical students, particularly during the years of their clinical training at the University of Jeddah. Our findings also showed that the COVID-19 pandemic had an impact on the students' financial, hygienic, and mental health, which led to increased depression and concern about visiting hospitals and providing care for patients, which ultimately prevented them from acquiring the necessary clinical skills.

## Introduction

On Jan 30, 2020, the World Health Organization (WHO) proclaimed coronavirus disease 2019 (COVID-19) to be a global public health emergency, and on March 11 2020 declared the outbreak as a pandemic [1,2]. The first incident in Saudi Arabia was announced on March 7 [2]. Afterward, the government responded quickly and effectively, beginning with a social media campaign advising people to stay at home and obey the Ministry of Health's recommendations [2]. Moreover, human civilization is likely to be at its most critical crossroads in this millennium, which has made a large number of the world's population isolated from their external surroundings due to the preventive measures imposed by the affected countries to limit the spread of the virus [3].

The COVID-19 pandemic has had significant ramifications for public institutions as it forced countries into lockdowns and dramatically altered their routine and rituals and many governments have established stay-at-home directives, which led to the shutdown of many colleges and universities. People from all around the world, including medical students, were affected by raising specific concerns for medical schools [4,5]. In the United Kingdom (UK), although many medical students are preparing for or participating in exams that need clinical exposure, COVID-19 had a significant impact, as multiple teaching hospitals in the UK have reported COVID-19 incidents, with several suspending medical and observership students from clinical attachments [6]. On March 17, 2020, due to the rapid spread of the pandemic in the United States of America, the Association of American Medical Colleges (AAMC) issued decisions and directives, which were the first in modern US history, to stop all clinical courses immediately [4]. Numerous difficulties with these decisions were reported at Alfaisal University's College of Medicine in Riyadh, Saudi Arabia, including problems with communication, student evaluation, technology use, online experience, pandemic-related anxiety or stress, time management, and technophobia [7]. A study was conducted among 40 UK medical schools to learn about medical students' perspectives on the use of online learning in facilitating medical education during the COVID-19 pandemic. The flexibility of online education platforms was referred to as one of the most attractive features. Whereas family distraction and a poor internet connection were two of the most commonly identified impediments to adopting online education platforms [8]. Moreover, a cross-sectional study on 418 undergraduate and postgraduate medical students from around the world was conducted, revealing that 96% of students went through difficulties with learning, including trouble with memorization, concentration, and making mistakes. In addition, 90% stated overall learning struggles during the pandemic in relation to the pre-pandemic era [9].

In regard to the mental aspect, a systematic review of 43 studies measuring psychiatric symptoms and morbidities associated with COVID-19 illustrates that the general public displayed decreased psychological well-being and increased scores of anxiety and depression in contrast to the pre-COVID-19 eras [10]. In order to protect themselves from the virus, individuals needed to adopt a routine of handwashing and disinfecting objects, as well as isolation and social distancing. A cross-sectional study conducted in March 2020 in Jordan, utilizing an online questionnaire to assess certain precautions that medical students were taking toward COVID-19, concluded that more than 80% of the participants took certain precautions as their main defense against COVID-19, such as social isolation, constant hand washing, and improved personal hygiene [11].

Universities and other educational institutions in Saudi Arabia, including medical learning facilities, were forced to adopt stronger regulations in order to maintain their instructional programs. Therefore, this article aims to highlight the impact of COVID-19 on the academic, as well as financial, psychological, and hygienic aspects on medical students in order to improve how universities handle such pandemics and enable students to continue and develop their own methods of training even under such circumstances.

## Materials and methods

Study design

It was an observational descriptive cross-sectional study. Conducted among medical students at the University of Jeddah, Jeddah, Saudi Arabia in 2022.

Eligibility and participants

Samples were taken randomly using a simple consecutive type of sampling from our target population of 350 medical students, who were currently enrolled at the time of the study's conduction.

Our inclusion criteria actively involved MBBS undergraduate students and interns at the University of Jeddah, Saudi Arabia who experienced the teaching process during the COVID-19 era. However, students who weren’t subjected to the medical educational system during the COVID-19 pandemic, and missing data are all excluded. Students are classified by their academic years (Starting from the second to the internship year).

Sample size

The sample size was calculated using the Raosoft calculator. We determined the sample size based on recent data, estimating the total number of medical students at the University of Jeddah to be 700. A sample size of 294 was needed to achieve a 95% confidence interval with a 5% margin of error. Since it was anticipated that fewer respondents would complete an online survey, the sample size was increased.

Settings and sources

Students filled out an e-questionnaire (Appendix 1). The questionnaire was modified from a pre-existing and valid version with the permission of the authors [12]. A few questionnaire questions regarding the students choosing what university they study at were omitted to make it fit our inclusion criteria.

The data collection period lasted from June to July 2022. Age, gender, academic year, residence area, university, and GPA were among the demographic variables assessed. We investigated the impact of the pandemic on academic components (we inquired about its impact on GPA, lectures, and the effect on professors, clinics, and rounds), hygienic behaviors, psychological stress, financial consequences, and the impact on elective training.

Ethical consideration

The study's application number, UJ-REC-067, was authorized by the Institutional Review Board (IRB) of the University of Jeddah. Consent was obtained at the beginning of the questionnaire and was also required to finish it.

Statistical analysis

The statistical analysis was done using Statistical Package for Social Sciences (SPSS) version 25 (IBM Corp., Armonk, NY, USA). The data were imported into SPSS after being inserted into a Microsoft Excel 2010 worksheet. The ordinal variables (e.g., the Likert Scale) were described using mean/median (IQR/SD), while the nominal variables (e.g., gender) were described using counts (frequency). The information acquired was summarized and organized into tables.

Missing data were not included in the total number of participants for each variable; thus, the percentages in the tables only represent the valid percentages. The mean of the items in each variable group was calculated to get a score of 10 points. This score was created to reflect the financial, hygienic, and academic implications of COVID-19. The higher this score, the larger the effect of COVID-19 on the aforementioned aspects. Scores are summarised and reduced into five classes: 1 and 2 are referred to as “strongly disagree”, 3 and 4 as ”disagree”, 5 and 6 are “Neutral”, “agree” is assigned for 7 and 8, and 9 and 10 are considered “strongly agree”.

The Chi-Square test was used to study the difference in the effect of the COVID-19 pandemic on academic achievement, mental health, and hygiene behaviors among gender and year of study (i.e., basic vs. clinical years). P values < 0.05 is considered to be statistically significant. All underlying assumptions were met unless otherwise indicated.

## Results

Demographics

Out of the 333 responses, males made up 174 (52.3%), while females made up 159 (47.7%). The age group 18-20 years (n=73, 21.9%) and 21-23 years (n=237, 71.2%) were the two most prevalent age groups. Moreover, most responders (n=307, 92.2%) were Jeddah residents. For a total GPA out of 5, most of the respondents had a range between 4-5 (n=241, 72.4%) (Table 1).

**Table 1:** General characteristics of the study participants.

Variable	Level	N (%)
Gender	Male	174 (52.3%)
	Female	159 (47.7%)
Age	18-20	73 (21.9%)
	21-23	237 (71.2%)
	24-26	23 (6.9%)
Academic year	2nd year student	102 (30.63%)
	3rd year student	129 (38.74%)
	4^th^ year student	59 (17.72%)
	5^th^ year student	24 (7.21%)
	6^th ^year student	12 (3.6%)
	Medical intern	7 (2.1%)
City of Living	Living in Jeddah	307 (92.2%)
	Living outside Jeddah	26 (7.8%)
GPA	4-5	241 (72.4%)
	3-4	79 (23.7%)
	<3	13 (3.9%)
Source of information about sanitization and overall hygiene	Social Media	150 (45%)
	WHO	122 (37%)
	Community	58 (17%)
	Other	3 (1%)

The academic effect of COVID-19

The students' median response to the question of whether the COVID-19 pandemic had a negative impact on their academic grades was 5 (IQR: 3-8) (Table 2), and there was no statistically significant difference between the groups as shown in Table 3. As for online teaching, 68.1% (n=227) and 55.8% (n=186) agreed or strongly agreed that “technical issues are online teaching difficult” and “teachers not being familiar with technology is one of the main issues facing online teaching”, respectively. Likewise, the majority (54%, n=180) agreed or strongly agreed that “Fluctuations in lecture timing is one of the downsides of Online teaching” and “The hands-on experience has suffered greatly” with a median of 7 (IQR=5-9). On the other hand, 143 (42.9%) of participants strongly agreed with “Recorded lectures are better than live lectures as they enable the student to set their own learning time” while a small minority of 17 (5.1%) strongly disagreed, and to that “The home environment is suitable for attending online lectures”, 117 (35.2%) strongly agreed and 31 (9.1%) strongly disagreed with a median of 7 (IQR=5-10) (Table 2). Furthermore, Table 3 illustrates a significant difference in terms of agreeing that “hands-on experience has suffered greatly”, of 7.3% between the males (n=88,50.6%) and females (n=92,57.9%) (P=0.002), and 22.4% difference between basic year (n=109,47.2%) and clinical year (n=71,69.6%) (P=0.001).

**Table 2:** The effect of the COVID-19 pandemic on the academic aspect

Variable	Strongly Disagree 1-2	Disagree 3-4	Neutral 5-6	Agree 7-8	Strongly Agree 9-10	Median (IQR)
Academic grades						
COVID-19 pandemic Affected Acdamic grades Negatively	78(23.4%)	51(15.3%)	75(22.5%)	65(19.5%)	64(19.3%)	5 (3-8)
Lecture and teachers						
Fluctuations in lecture timing is one of downsides of Online teaching	33(9.9%)	26(7.8%)	94(28.2%)	78(23.4%)	102(30.6%)	7 (5-9)
The hands-on experience has suffered greatly	15(4.5%)	26(7.8%)	112(33.6%)	85(25.5%)	95(28.5%)	7 (5-9)
Recorded lectures are better than live lectures as they enable the student to set their own learning time.	17(5.1%)	34(10.2%)	57(17.1%)	82(24.7%)	143(42.9%)	8 (6-10)
Personalized feedback from training centers has a positive impact on students and raises the motivation levels of the students, unlike online teaching where students' feedback is limited.	26(7.85%)	39(11.7%)	83(24.9%)	84(25.2%)	101(30.35%)	7 (5-9)
Teachers not being familiar with technology is one of the main issues facing online teaching.	30(9%)	44(13.2%)	73(22%)	76(22.8)	110(33%)	7 (5-10)
Technical issues like poor WiFi connection and lack of computer or mobile devices make online teaching difficult.	16(4.9%)	30(9%)	60(18%)	87(26.1%)	140(42%)	8 (6-10)
The home environment is suitable for attending online lectures.	31(9.3%)	26(7.8%)	65(19.5%)	94(28.2%)	117(35.2%)	7 (5-10)
Clinics and Rounds						
The Rounds/clinics became less informative.	20(6%)	32(9.6%)	146(43.8%)	72(21.7%)	63(18.9%)	6 (5-8)
The rounds/clinics limit the number of students present.	18(5.4%)	22(6.6%)	129(38.7%)	80(24%)	84(25.3%)	6 (5-9)
I feel the clinics/rounds have been affected during COVID-19 negatively.	13(3.9%)	23(6.9%)	116(34.8%)	69(20.8%)	112(33.6%)	7 (5-10)
There are fewer patients to examine.	18(5.4%)	30(9%)	147(44.1%)	56(16.8%)	82(24.7%)	6 (5-8)
Patients in the hospital became reluctant to be cooperative with students.	20(6%)	26(7.8%)	161 (48.3%)	65(19.6%)	61(18.3%)	6 (5-8)
The Rounds and clinics became shorter	18(5.4%)	23(6.9%)	125(37.5%)	76(22.8%)	91(27.3%)	7 (5-9)

**Table 3:** Differences in the effect of COVID-19 pandemic on academic, financial, mental health and hygiene behaviors among gender and year of study *a score of 7 or more is considered as “agree”

Variable	Level	N (%) of participants who agreed *	P-value
COVID-19 pandemic negatively affected academic grades	Male	66 (37.9%)	0.240
	Female	63 (39.6%)	
	Basic Years	88 (38.1%)	0.217
	Clinical Years	41 (40.2%)	
The clinics /rounds had been negatively affected during COVID-19	Male	94 (54%)	0.503
	Female	87 (54.7%)	
	Basic Years	101 (43.7%)	<0.001
	Clinical Years	80 (78.4%)	
The hands – on experience has suffered greatly	Male	88(50.6%)	0.002
	Female	92 (57.9%)	
	Basic Years	109 (47.2%)	0.001
	Clinical Years	71 (69.6%)	
The pandemic affects future specialty choice	Male	40 (23%)	0.216
	Female	46 (28.9%)	
	Basic Years	54 (23.4%)	0.124
	Clinical Years	32 (31.4%)	
The pandemic changed how we consider our budget carefully	Male	78 (44.8%)	0.934
	Female	72 (45.3%)	
	Basic Years	101 (43.7%)	0.170
	Clinical Years	49 (48%)	
COVID-19 impacted mental health	Male	69 (39.7%)	0.012
	Female	85 (53.5%)	
	Basic Years	109 (47.2%)	0.605
	Clinical Years	45 (44.1%)	
Always wash hands for least 20s and in an appropriate way	Male	118 (67.8%)	0.466
	Female	113 (71.1%)	
	Basic Years	155 (67.1%)	0.161
	Clinical Years	76 (74.5%)	
Always sanitize hands before touching eyes, nose, mouth	Male	110 (63.2%)	0.604
	Female	96 (60.4%)	
	Basic Years	134 (58%)	0.070
	Clinical Years	72 (70.6%)	
Became more aware of sensitizing medical equipment after examining each patient	Male	122 (70.1%)	0.345
	Female	115 (72.3%)	
	Basic Years	155 (67.1%)	0.001
	Clinical Years	82 (80.4%)	

In terms of clinics and rounds, only 10.8% (n=36), 12% (n=40), 12.3% (n=41), 13.8% (n=46), 14.4% (n=48), and 15.6% (n=52) students disagreed or strongly disagreed with the statements “I feel the clinics/rounds have been affected negatively during COVID-19”, “the clinics/rounds limit the number of students present”, “the clinics/rounds became shorter”, “patient in the hospital became less cooperative”, “there are fewer patients to examine”, and “ the clinics/rounds became less informative, respectively (Table 2).

The effect of COVID-19 on elective training and mental health

One hundred five students (31.5%) had electives during the COVID-19 pandemic. Of the 105, 41 (39%) had it online and 64 (61%) had it at training centers. Regarding the elective place, a total of 94 (89.5%) had it in Saudi Arabia, and 11 (10.5%) had it elsewhere. COVID-19 had an impact on 154 (46.2%) students’ mental health. Of those 154, a total of 111 (72.1%) developed anxiety or depression, while the remaining 43 (27.9%) denied having either. A third (34.5%) of people whose mental health was affected developed an obsession with getting the illness (Table 4). There is a significant difference in the number of people affected from each gender, as more than half (53.5%) of the females were mentally affected by the COVID-19 pandemic, compared to only 39.7% of males (P=0.012) (Table 3).

**Table 4:** The effect of COVID-19 pandemic on elective and mental aspects.

Variable	Yes (%)		No (%)
Had an elective during the COVID-19 pandemic	105 (31.5%)		228 (68.5%)
Place of elective (n=105)	Saudi Arabia 94 (89.5%)	elsewhere 11 (10.5%)	
Type of elective (n=105)	In training center 64 (61%)	Online 41 (39%)	
COVID-19 Pandemic impacted mental health	154 (46.2%)		179 (53.8%)
Became anxious or depressed (n=154)	Anorexic or depressed 111 (72.1%)	Not anorexic nor depressed 43 (27.9%)	
Became obsessed with contracting the disease (n=154)	Obsessed with contracting the disease 53 (34.5%)	Not obsessed with contracting the disease 101 (65.5%)	

The effect of COVID-19 on hygiene behaviors and financial aspects

As shown in Table 1, social media (n=150, 45%) was the most used source of information during the COVID-19 pandemic, with World Health Organization (WHO) (n=122, 36.6%) being the second. The medians regarding the COVID-19 pandemic impact on hygienic behaviors were 7 or more, with 231 (69.3%) agreeing or strongly agreeing with “Always wash your hands for at least 20 seconds and in an appropriate way”, and the majority (n=176, 52.8%) strongly agreed with “Became more aware of sanitizing medical equipment after examining each patient”. In terms of patient awareness, the majority (n=234,70.2%) either agreed or strongly agreed on “Helped in raising hygiene awareness of people around me”, and 66.9% (n=223) agreed or strongly agreed with “Noticed a rising in patients’ awareness of self-hygiene” (Table 5). Regarding the above-mentioned points, Table 3 illustrated a male-to-female ratio of less than 5%, yet there is a difference between basic and clinical students of 7.5% to 13.3%.

**Table 5:** The effect of COVID-19 pandemic on sanitization and hygiene behaviors, and financial aspects.

Variable	Strongly Disagree 1-2	Disagree 3-4	Neutral 5-6	Agree 7-8	Strongly Agree 9-10	Median (IQR)
Hygienic aspect						
Always wash hands for least 20 s and in an appropriate way	8(2.4%)	31(9.4%)	63(18.9%)	95(28.5%)	136(40.8%)	8 (6-10)
Became more aware of sensitizing medical equipment after examining each patient	4(1.3%)	15(4.5%)	77(23.1%)	61(18.3%)	176(52.8%)	9 (6-10)
Sanitize hands before touching eyes, nose, or mouth	12(3.7%)	35(10.5%)	80(24%)	80(24%)	126(37.8%)	7 (5.5-10)
Helped in raising hygiene awareness of people around me	8(2.5%)	22(6.6%)	69(20.7)	89(26.7%)	145(43.5%)	8 (6-10)
Noticed a raising in patients’ awareness of self-hygiene	5(1.6%)	25(7.5%)	80 (24%)	88(26.4%)	135(40.5%)	8 (6-10)
Financial aspect						
The Pandemic made me budget more carefully	30(9%)	35(10.5%)	118(35.5%)	74(22.2%)	76(22.8%)	6 (5-8)
household suffered financially	60(18%)	66(19.8%)	93(27.9%)	68(20.4%)	46(13.9%)	5 (3-7)
Used different methods to lower transportation cost	54(16.2%)	47(14.1%)	121(36.4%)	64(19.2%)	47(14.1%)	5 (4-7)

In regards to financial aspects, 150 (45%) agreed or strongly agreed with “The Pandemic made me budget more carefully”, while 219 (65.7%) ranged from strongly disagree to neutral in regards to “household suffered financially” (Table 5). Both males (n=78, 44.8%) and females (n=72, 45.3%) showed similar results with regard to “the pandemic made me think of my budgeting more carefully”. In terms of the pandemic's impact on future specialty choices, clinical year students (n=32, 31.4%) showed a higher percentage at an increase of 8% over basic year students (n=54, 23.4%) (P=0.124) (Table 3).

## Discussion

Academic aspect

Influences of many factors on the performance of medical students are being evaluated, where many studies assessed the premedical, academic, maturity, and familial support and its relation to the medical students' performance [13], this study indicates that online teaching may benefit or undermine the education system in the COVID-19 era. For instance, technical difficulties, changes in lecture timing, patients being less cooperative, a lack of hands-on experience, and teachers' lack of expertise with technology are some drawbacks that students experienced. However, recorded lectures and suitable home environments made online teaching more favorable for students. A study in Oman that evaluated various factors influencing clinical teaching indicates that insufficient technologies and audiovisual resources were concerns for more than half of the students [14]. This might be the outcome of their first exposure to technology throughout medical education. As to previous research on medical schools in the area, 41.8% of faculty and students said they had little to no experience with online teaching and learning [7].

It has been hypothesized that the impact was greater during the clinical years than during the basic years. Online classes have proven to be a fantastic option for theoretical courses like basic sciences, according to a survey that was done to assess how students felt about them. They are nevertheless inappropriate for clinical disciplines like clinical skills [15]. 

Mental aspect

The numbers indicate that medical students were susceptible to poor mental health during the COVID-19 pandemic, with females being more severely affected. This may be brought on by a decline in interpersonal communication during the COVID-19 era, which increases the likelihood of anxiety disorders [16]. Moreover, this mental deterioration includes concerns in regard to studies, or having a sick family member, and may reach a percentage of 68% [17]. According to a study by Wenjun Cao, living away from one's parents and being a student in a rural location are both positively correlated with anxiety symptoms. Moreover, It is suggested that in order to give college students timely, high-quality psychological assistance focused on crises, the government, and educational institutions should work together to find a solution to this issue [18].

Hygienic aspect

Although no difference between males and females, basic-year and clinical-year students, the overall results illustrate that the COVID-19 pandemic had a positive effect in regard to the hygienic aspect of the participants. More than 60% agreed with washing their hands in a proper way, being aware of sanitizing medical equipment after use, sanitizing hands before touching one’s face, raising awareness of the surrounding people, and noticing a rise in patients’ self-hygiene. This high number could be due to the fear of contracting the disease or from the country’s efforts to educate the public on proper hygiene in order to avoid being infected. According to a cross-sectional study conducted on Jouf University students in 2020, the Saudi Ministry of Health’s education program was potent in terms of raising awareness and educating the public in regard to COVID-19 [19]. Similar results can be found in a 2020 cross-sectional study conducted in Turkey which states that the hygienic practices of academic and student participants have increased during the COVID-19 pandemic [20].

Financial aspect

The financial aspect wasn’t affected as severely as other sections, since only around one-third of the participants had their household suffer with respect to money and explored different options in order to lower transport costs. This might be because the working members of those families weren’t in jobs that required real-life interactions and could work from home during the lockdown. Another explanation could be that the lockdown allowed for some money-saving in terms of gasoline for the car and spending on activities outside of the house like malls and arcades. Around 45% of the participants stated that they had to start budgeting more carefully during the lockdown, which is close in value in relation to a 2020 cross-sectional study conducted on medical students in Jordanian universities which concluded that almost half of the participants were budgeting more carefully during the pandemic [12].

Limitations

Almost all of the University of Jeddah's medical students participated in this study, which is the first to investigate how the COVID-19 pandemic has affected medical students there. However, it is important to emphasize that our study had a number of limitations; since this survey only included medical students from the University of Jeddah and did not take into account medical students from other universities, and other factors affecting academic performance were not included which may lead to a less valid assessment of academic performance. Moreover, this study only evaluated academic, financial, hygienic, and specialty/elective aspects. Therefore, further research is required, involving students and educators from many universities, to go into greater detail about the effects of the COVID-19 pandemic and possible outcomes in other aspects.

## Conclusions

The COVID-19 pandemic had a significant impact on medical students' academic growth at the University of Jeddah, with a more pronounced impact during clinical years. Despite this, several advantages of online instruction were noted, such as the flexibility of online classrooms. Additionally, the COVID-19 pandemic had an effect on the students' financial, mental, and hygienic well-being, making them unhappier and reluctant to visit hospitals and provide treatment for patients, according to our findings. Our analysis thus offered workable remedies to current negative consequences and potential future issues of a similar nature. Included in this are creating awareness campaigns on proper hand washing and how to handle potential infectious crises in the future.

## Appendices

**Table 6:** Questionnaire used for data collection

Question	Answers
1) Gender	Male, Female
2) Age	18-20, 21-23, 24-26
3) Your Current Academic Year	1st year, 2nd year, 3rd year, 4th year, 5th year, 6th year, Medical Intern
4) Where do you live	Jeddah, Other ------
5) What is your current GPA?	<3, 3-4,, >4
6) COVID-19 pandemic affected your academic grades negatively	Disagree 1 2 3 4 5 6 7 8 9 10 agree
7) Fluctuations in lectures' timing unlike fixed schedule of training centers is one of the downsides of online teaching.	Disagree 1 2 3 4 5 6 7 8 9 10 agree
8) Personalized feedback from training centers has a positive impact on students and raising the motivation levels of the students unlike online teaching where students feedback is limited.	Disagree 1 2 3 4 5 6 7 8 9 10 agree
9) Teachers not being familiar with technology is one of main issues facing online teaching.	Disagree 1 2 3 4 5 6 7 8 9 10 agree
10) Recorded lectures are better than live lectures as it enables the student to set their own learning time.	Disagree 1 2 3 4 5 6 7 8 9 10 agree
11) Technical issues facing online teaching as poor WiFi connections and lack of computer or mobile devices makes it difficult.	Disagree 1 2 3 4 5 6 7 8 9 10 agree
12) I feel the clinics/rounds have been affected during Covid-19 negatively.	Disagree 1 2 3 4 5 6 7 8 9 10 agree
13) The hands-on experience has suffered greatly.	Disagree 1 2 3 4 5 6 7 8 9 10 agree
14) The rounds/clinics have become shorter.	Disagree 1 2 3 4 5 6 7 8 9 10 agree
15) There are fewer patients to examine.	Disagree 1 2 3 4 5 6 7 8 9 10 agree
16) Patients in the hospital became reluctant to be cooperative with students.	Disagree 1 2 3 4 5 6 7 8 9 10 agree
17) The rounds/clinic limits the number of students present.	Disagree 1 2 3 4 5 6 7 8 9 10 agree
18) The rounds/clinics became less informative.	Disagree 1 2 3 4 5 6 7 8 9 10 agree
19) You always wash your hands for at least 20 seconds and in an appropriate way.	Disagree 1 2 3 4 5 6 7 8 9 10 agree
20) You sanitize your hands before touching your eyes, nose, or mouth.	Disagree 1 2 3 4 5 6 7 8 9 10 agree
21) You are more aware of sanitizing your medical equipment's after examining each patient.	Disagree 1 2 3 4 5 6 7 8 9 10 agree
22) You helped in raising hygiene awareness of people around you.	Disagree 1 2 3 4 5 6 7 8 9 10 agree
23) You noticed a raise in patients’ awareness of self-hygiene.	Disagree 1 2 3 4 5 6 7 8 9 10 agree
24) Your source of information about sanitization and overall hygiene is?	Social media, WHO, Community
25) Did covid-19 impact your mental health?	Yes, No, It didn’t affect my mental health
26) If impacted your mental health, did you become more anxious and depressed?	Yes, No, It didn’t affect my mental health
27) If yes impacted your mental health, Did you become obsessed with contracting the disease?	Disagree 1 2 3 4 5 6 7 8 9 10 agree
28) Did Covid-19 affect you financially?	Yes, No
29) The pandemic made you think of your budgeting more carefully.	Disagree 1 2 3 4 5 6 7 8 9 10 agree
30) Your household suffered financially.	Disagree 1 2 3 4 5 6 7 8 9 10 agree
31) You used different means to lessen your transportation expenses.	Disagree 1 2 3 4 5 6 7 8 9 10 agree
32) If you’re from outside of Jeddah, did you return to your Home at the beginning of the pandemic?	Yes, No, I’m from Jeddah
33) You found your home environment suitable for practicing online lectures ?	Disagree 1 2 3 4 5 6 7 8 9 10 agree
34) Did you have your elective training?	Yes, No
35) If yes, did you take it in Saudi Arabia or elsewhere?	Yes in Saudi Arabia, No elsewhere,
36) Was The elective training online or in training centres?	Online, In training centres, Not Applicable
37) The pandemic affected your selection of elective location.	Disagree 1 2 3 4 5 6 7 8 9 10 agree
38) The pandemic affected your residency/specialty choice.	Yes, No
